# Inadvertent retention of Descemet membrane after penetrating keratoplasty for pseudophakic bullous keratopathy

**DOI:** 10.1093/jscr/rjac298

**Published:** 2022-08-31

**Authors:** Alejandro Ontiveros-Holguín, Jorge Pacheco-Padrón

**Affiliations:** Department of Medicine, Universidad Autónoma de Durango Campus Chihuahua, Chihuahua, Chihuahua, Mexico; Department of Ophthalmology, Hospital Angeles Chihuahua, Chihuahua, Chihuahua, Mexico

## Abstract

A retained Descemet membrane pertains to a type of retrocorneal membrane—a well-known yet rare complication of penetrating keratoplasty. We present a case of retained Descemet membrane after penetrating keratoplasty for pseudophakic bullous keratopathy. A 71-year-old woman presented to the ophthalmology clinic for loss of visual acuity. The previous year she had undergone phacoemulsification on both eyes, resulting in pseudophakic bullous keratopathy in the right eye; an uneventful penetrating keratoplasty was performed on the affected eye. The following day at follow-up, an undulated retrocorneal membrane was discovered on slit-lamp examination: corresponding to a retained Descemet membrane. A satisfactory descemetorhexis was performed. Timely diagnosis and intervention allowed for a remarkable outcome, with a best-corrected visual acuity of 20/50 OD with contact lens use.

## INTRODUCTION

Pseudophakic bullous keratopathy is the development of irreversible corneal edema following cataract surgery due to a loss of corneal endothelial cells by surgical trauma of the endothelium [1]. Consequent to this lesion, an inrush of aqueous humor into the corneal stroma takes place, forming blister-like lesions or bullae in addition to corneal edema, all of which contribute to corneal opacification [1]. As such, patients with this condition present with an important decrease in visual acuity, epiphora and even ocular pain related to corneal nerve stretching or ruptured bullae [1]. Management includes a trial of topical hypertonic agents, anti-inflammatory agents, among other drugs. If the above fails, corneal grafting is warranted, which to date remains the gold standard therapy for pseudophakic bullous keratopathy [1].

## CASE REPORT

A 71-year-old woman presented for the first time to the ophthalmology clinic for loss of visual acuity. She had diabetes mellitus type 2 and was otherwise healthy. Slit-lamp examination exposed the presence of severe edema affecting the central area of the cornea along with bullae in the right eye (OD). Her best-corrected visual acuity (BCVA) was counting fingers at 1 meter OD and 20/40 left eye. The previous year, she had undergone phacoemulsification on both eyes, leading to endothelial decompensation in the right eye with the appearance of pseudophakic bullous keratopathy shortly thereafter.

**Figure 1:**
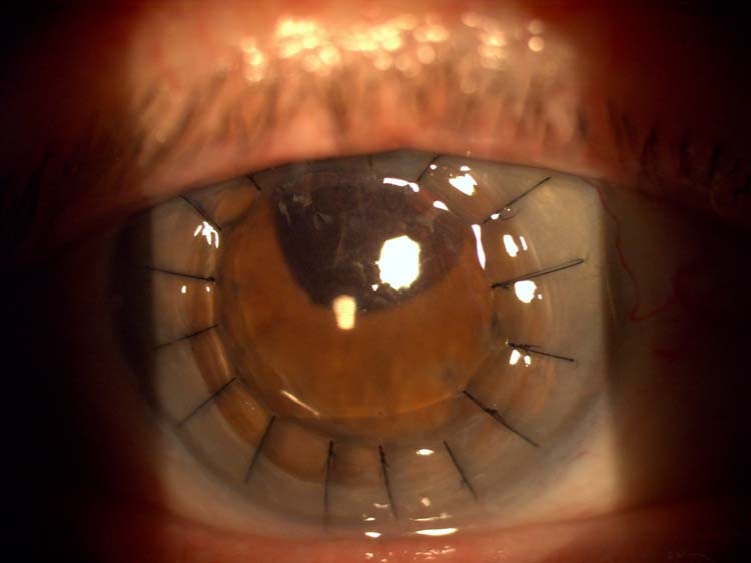
Slit-lamp photograph of the right eye the day after penetrating keratoplasty.

**Figure 2:**
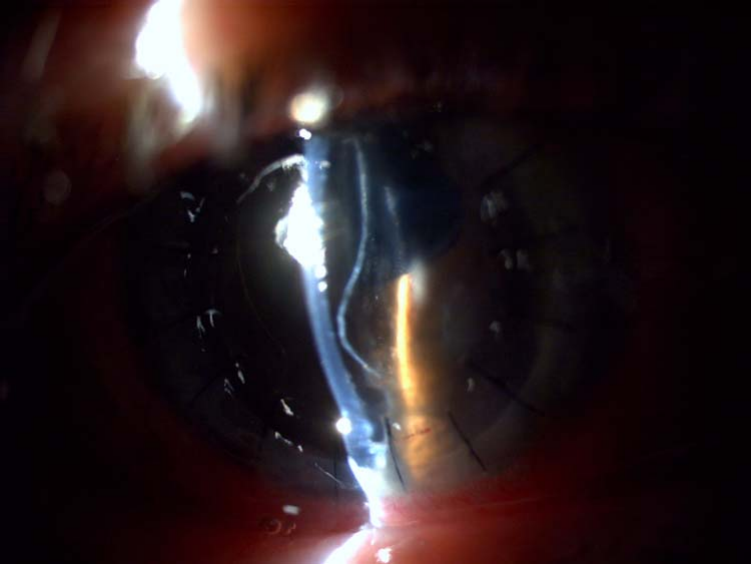
Slit-lamp visualization of a wavy retained Descemet membrane with supernumerary anterior chamber formation in the right eye on the day following penetrating keratoplasty.

**Figure 3:**
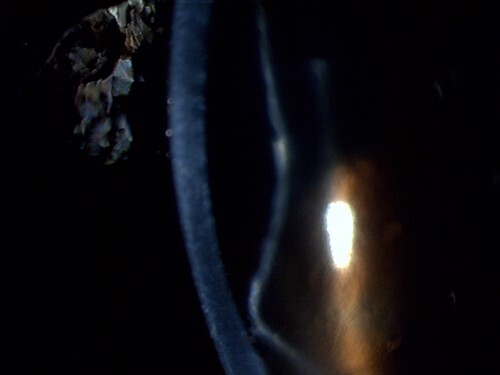
Slit-lamp photograph zoomed in on the cross-section of the cornea and the retained Descemet membrane in the right eye.

Owing to this circumstance, an uneventful penetrating keratoplasty was performed under general anesthesia. The following day at follow-up visit, slit-lamp examination revealed an undulated retrocorneal membrane: corresponding to a retained Descemet membrane (Figs 1–3). Aside from this membrane, everything else was unremarkable upon examination. It was then decided to remove the retained membrane to preserve graft viability. An initial neodymium-doped yttrium aluminum garnet (Nd:YAG) laser membranotomy was performed, followed by viscodissection of the retrocorneal membrane and concluded with a manual descemetorhexis. No further complications arose and the patient's final BCVA was 20/50 OD with contact lens use 1 year later.

## DISCUSSION

A retained Descemet membrane pertains to a type of retrocorneal membrane—a well-known yet rare complication of penetrating keratoplasty—caused by an incomplete removal of the host cornea and in which the retained Descemet membrane creates a duplication of the anterior chamber behind the corneal graft [2]. This retained membrane can lead to corneal graft failure by a progressive endothelial cell loss [3].

Several types of retrocorneal membranes have been described in the context of penetrating keratoplasty, each with its own etiopathogenesis, but a retained Descemet membrane is the only one that will show a clear cornea with a wavy retrocorneal membrane on the next day after penetrating keratoplasty [2]. This retained membrane may be due to a poorly attached Descemet membrane secondary to longstanding corneal edema, viscodissection of the host Descemet membrane before trephination or simply by an unintended incomplete trephination [2, 4]. The diagnosis is clinical: a double anterior chamber created by an undulated layer behind the cornea can be observed with the slit-lamp at the first follow-up visit [2].

In this case, typical findings on slit-lamp examination allowed for a brief diagnosis of retained Descemet membrane. Membrane excision was deemed appropriate to avoid further complications and provide the best outcome for our patient. Other reports have successfully used triamcinolone acetonide [5] and trypan blue [6] as an aid in the excision of this added membrane. Timely diagnosis and intervention led to a remarkable outcome, with a BCVA of 20/50 OD.

As proposed by Mihail *et al.*, the best way to avert a retained Descemet membrane is to attempt to reach the iris with forceps and avoid relying on aqueous humor leakage, as this does not indicate a complete cutout of Descemet membrane [2].

## PATIENT CONSENT

Consent was not needed as no personal identifying information is disclosed in this report.

## AUTHORSHIP

All authors meet the International Committee of Medical Journal Editors (ICMJE) criteria for authorship.

## CONFLICT OF INTEREST STATEMENT

The authors certify that they have no involvement in any organization with any financial or non-financial interest in the subject matter discussed in this case.

## FUNDING

No funding or grant support was required.

## References

[1] Pricopie S , Istrate S, Voinea L, Leasu C, Paun V, Radu C. Pseudophakic bullous keratopathy. Rom J Ophthalmol 2017;61:90–4. 29450379 10.22336/rjo.2017.17 PMC5710027

[2] Mihail Z , Alina-Cristina S, Speranta S. Retrocorneal membranes after penetrating keratoplasty. Rom J Ophthalmol 2015;59:230–4. 29450312 PMC5712944

[3] Choi JS , Oh JY, Wee WR. A case of corneal endothelial deterioration associated with retained Descemet’s membrane after penetrating keratoplasty. Jpn J Ophthalmol 2009;53:653–5. 20020249 10.1007/s10384-009-0737-9

[4] McVeigh K , Cornish KS, Reddy AR, Vakros G. Retained Descemet’s membrane following penetrating keratoplasty for Fuchs’ endothelial dystrophy: a case report of a post-operative complication. Clin Ophthalmol 2013;7:1511–4. 23901260 10.2147/OPTH.S45161 PMC3726520

[5] Tyring A , Chang O, Jung H. Triamcinolone acetonide-assisted descemetectomy for retained Descemet membrane. COP 2018;9:227–31. 10.1159/000487706 PMC5903106 29681841

[6] Sinha R , Vajpayee RB, Sharma N, Titiyal JS, Tandon R. Trypan blue assisted descemetorhexis for inadvertently retained Descemet’s membranes after penetrating keratoplasty. Br J Ophthalmol 2003;87:654–5. 12714422 10.1136/bjo.87.5.654 PMC1771681

